# Parental stress and adjustment in the context of rare genetic syndromes: A scoping review

**DOI:** 10.1177/1744629521995378

**Published:** 2021-04-19

**Authors:** Jacqueline Fitzgerald, Louise Gallagher

**Affiliations:** 8809 Trinity College Dublin, Ireland; 8809 Trinity College Dublin, Ireland; Children Health Ireland at Tallaght Hospital, Ireland; Cherry Orchard Hospital, Ireland

**Keywords:** rare chromosomal abnormalities, genetic syndromes, parent stress, parent wellbeing, review

## Abstract

Chromosomal abnormalities are now considered a common cause of intellectual disability. With increased genetic testing, phenotyping and technological advancements, many new syndromes have been identified. This review sought to explore parental stress and adjustment in the context of rare genetic syndromes to evaluate their clinical impact. A systematic review of English peer-reviewed literature across three databases (PsycINFO, Medline, CINAHL) was completed and 69 articles were included. Parents of children with rare genetic syndromes experienced greater distress relative to other disabilities. Differences in parental wellbeing were syndrome-specific relative to ASD thus demonstrating the need to consider the contribution of syndrome-specific phenotypes. Child emotional and behavioural difficulties were the most consistent predictor of parental distress. Research reflecting other factors such as physical health, syndrome-specific behaviours, benefit finding and, parental appraisal in the context of a rare genetic aetiology is required in order to support parental adjustment in these conditions.

## Introduction

Rare conditions are characterised by their relatively low prevalence of less than 200,000 people total in the United States and 1 in 2,000 people in the European Union (NIH, 2017). A recent study found that rare conditions impact 3.5–5.9% of the population, which translates to approximately 18–30 million people in Europe (Nguengang Wakap et al., 2020) and estimates have extended to 350 million people worldwide (Tambuyzer et al., 2020). Structural chromosomal abnormalities and single genetic disorders account for at least 72% of these conditions (Nguengang Wakap et al., 2020). Due to technological advancements in cytogenetic testing, chromosomal abnormalities have been recognised as a common cause of intellectual disability, explaining up to 15% of cases (Michelson et al., 2011; van Karnebeek et al., 2005). Detailed clinical and genetic characterisation has resulted in the description of many syndromes associated with intellectual disability such as Prader-Willi syndrome, Angelman syndrome, Williams syndrome, Smith-Magenis syndrome and DiGeorge syndrome (also known as velocardiofacial syndrome or 22q11.2 deletion syndrome) (Vissers and Stankiewicz, 2012). Chromosomal microarrays are now recommended as a first-tier diagnostic test for individuals with unexplained developmental delay, intellectual disability, autism spectrum disorders (ASD) and multiple congenital anomalies due to 10–20% diagnostic yield (Battaglia et al., 2013; Manning and Hudgins, 2010; Miller et al., 2010). With this testing and on-going development of genetic technologies, new syndromes will likely continue to be identified (Bejjani and Shaffer, 2008).

While this technology brought a surge in research attempting to elucidate behavioural phenotypes associated with rare intellectual disability syndromes (Fehr et al., 2010; Martens et al., 2008; Whittington et al., 2004; Williams, 2010), families of children with rare genetic syndromes have been considered less frequently. The majority of family research has focused on intellectual disability irrespective of aetiology or has focused on more common conditions linked with intellectual disability such as autism spectrum disorder (ASD) or Down syndrome (Bonis, 2016; Fairthorne et al., 2016; Hill and Rose, 2009; Phillips et al., 2017). This review aims to explore risk and protective factors associated with having a child with a rare genetic syndrome in order to inform future research, healthcare policy and to provide optimal psychosocial resources and family-centred care.

Research has predominantly focused on challenges or adverse outcomes such as stress, burden, strain and mental health difficulties experienced by parents of children with a disability. Parents reported greater stress and psychological difficulties such as anxiety and depression with having a child with a disability relative to those without a developmental disability or relative to the general population (Patton et al., 2018; Seymour et al., 2013; Totsika et al., 2011; Zablotsky et al., 2013b). Studies indicated that parental distress was associated with various factors including coping strategies, child adaptive behaviour, child maladaptive behaviour and child emotional difficulties (Firth and Dryer, 2013; Hill and Rose, 2009; Minnes et al., 2015; Totsika et al., 2011; Zablotsky et al., 2013a). Furthermore, families who have a child with a disability are also more likely to experience social isolation, lifestyle limitations and financial strain (Burton-Smith et al., 2009; Cidav et al., 2012; Griffith et al., 2012; Johnson et al., 2006; Schaaf et al., 2011).

There is a growing body of research that highlights the possibility of positive psychological change or growth as a result of adversity (Linley and Joseph, 2005). Researchers increasingly appreciate the need to move beyond the parental outcomes of adversity and explore how positive aspects contribute to parental adjustment in the context of having a child with a disability (Beighton and Wills, 2017; Blacher and Baker, 2007; Greer et al., 2006; Larson, 2010; Resch et al., 2012; Vilaseca et al., 2013). The importance of expanding parental research beyond the outcome measure of stress has also been noted in the study of genetic syndromes (Hodapp and Dykens, 2012). Increased personal strength, growth and fulfilment have been reported by parents of children with a disability (Beighton and Wills, 2017; Greer et al., 2006; Rapanaro et al., 2007; Vilaseca et al., 2013). Blacher and Baker also demonstrated that positive aspects of parents mediated the relationship between child behaviour difficulties and parental stress (Blacher and Baker, 2007). These studies highlight the importance of considering both adverse outcomes as well as positive aspects of parenting in order to explore parental adjustment in rare genetic syndromes.

The current review was primarily informed by two theoretical models; a Model of Stress in Families of Children with Developmental Disabilities (Perry, 2005) and the Resiliency Model of Family Stress and Adjustment (McCubbin and McCubbin, 1993) in order to capture both parental stress and adjustment. Perry (2005) described stressors as directly related to the child (child characteristics) or not directly attributable to the child (other life stressors). In this model, these stressors are moderated by both family resources (parent personal resources and family system resources) and supports outside of the immediate family (informal social supports, health care services) to evoke both positive and negative parental outcomes (Perry, 2005). The Resiliency Model of Family Stress and Adjustment also highlighted the role of family factors such as vulnerability, functioning, resources, appraisal and coping strategies in mitigating the effects of a stressor such as having a child with a disability on adjustment within families (McCubbin and McCubbin, 1993). Thus, this review aims to explore child, parent, family and contextual factors associated with parental stress and adjustment in the context of rare genetic syndromes.

The receipt of a genetic diagnosis is an experience unique to parents of children with genetic syndromes and the journey to diagnosis is often challenging and complex (Anderson et al., 2013; Ashtiani et al., 2014; Lingen et al., 2016). Parents report uncertainty in terms of prognosis due to the low prevalence and relatively recent clinical recognition of their child’s rare condition (Graungaard & Skov, 2007; Whitmarsh et al., 2007). Furthermore, the low occurrence of rare genetic syndromes poses challenges for elucidating accurate clinical and phenotypic information, and significant phenotypic variability within genomic syndromes has been highlighted (Girirajan and Eichler, 2010). Perceived uncertainty is recognised to negatively influence parental cognitive appraisal of their child’s condition (Folkman and Greer, 2000; Madeo et al., 2012) and increase parental psychological distress (Chaney et al., 2016; Perez et al., 2020). In addition, phenotypes of rare syndromic conditions are multifaceted and often include physical, cognitive, emotional and behavioural challenges (Fehr et al., 2010; Martens et al., 2008; Whittington et al., 2004; Williams, 2010), and thus may require significant caregiver input. Parents have also described the genetic diagnosis as a validation of their experience (Makela et al., 2009) thus highlighting possible benefits of diagnosis in terms of parental wellbeing. These additional complexities highlight the need to consider the role of genetic aetiology in parent and family research in the context of intellectual disabilities.

## Method

### Review approach

Scoping reviews use transparent and rigorous methods to synthesise research in order to clarify concepts, evidence and identify gaps in the literature (Arksey and O’Malley, 2005). Whereas systematic reviews attempt to answer a clearly defined research question and critically evaluate the quality of included literature, scoping reviews aim to provide a systematic overview of a broad topic (Peterson et al., 2017; Pham et al., 2014). Scoping reviews have previously been used to explore family wellbeing in disability (Lunsky et al., 2014; Tint and Weiss, 2016). The current scoping review of parental stress and adjustment in rare genetic syndromes was completed in line with PRISMA scoping review (PRISMA-ScR) guidelines (Tricco et al., 2018).

### Research procedure

A comprehensive review of three databases (PsycINFO, Medline, CINAHL) was completed to identify relevant articles. The search extended from the origin date of each database to March 2020. Peer-reviewed quantitative, qualitative and mixed methods articles written in English were included. All searches included a combination of keywords relevant to parents (e.g. parent, mother, father, maternal, paternal, caregiver, family), stress/adjustment (e.g. stress, burden, quality of life, resilience, adjustment, adaptation), rare genetic syndromes (e.g. syndrome, rare genetic, deletion, duplication) limited to the abstract search field and intellectual disability (e.g. intellectual disability, learning disability) in the full text field. Search terms were truncated where appropriate to maximise the search outcomes. A follow up search was conducted with specific genetic syndromes identified in the first search (e.g. Rett syndrome, Fragile X syndrome, Angelman syndrome, Smith-Magenis syndrome, Williams syndrome). All searches were performed between January and March 2020.

### Inclusion and exclusion criteria

Articles were selected based on the following inclusion criteria: (a) studies presented original research published in a peer-reviewed English-language journal, (b) study participants included parents of individuals with a rare genetic syndrome (prevalence less than 1 in 2000), and (c) the topic of study incorporated the general stress or adjustment of parents of individuals with a rare genetic syndrome. The exclusion criteria were as follows: (a) studies that pooled parents with other family members or groups, (b) studies that did not distinguish rare genetic syndromes from common syndromes (prevalence greater than 1 in 2000), syndromes not commonly associated with intellectual disability or from other physical disabilities, (c) review articles and, (d) articles that specifically focused on parenting programmes or interventions. The presence or absence of the intellectual disability phenotype as well as syndrome prevalence was checked against rare disease online resources (NORD, NIH Genetic and Rare Diseases Information Center and Orphanet) (Hogan Smith, 2017).

### Data extraction

Data extracted from full texts consisted of, the author(s), year of publication, title of the study, country of origin, demographics, methodology, and key findings (e.g. themes for qualitative studies and statistically significant results for quantitative studies).

### Data synthesis

A deductive analysis of relevant findings from each full text was performed. Results pertinent to the research question were then coded and grouped by their codes. Key findings were subsequently categorised into five key areas: (a) parental outcomes (b) child-related factors, (c) parent-related factors, (d) family-related factors and, (e) contextual factors.

## Results

### Search results

**Figure 1.**
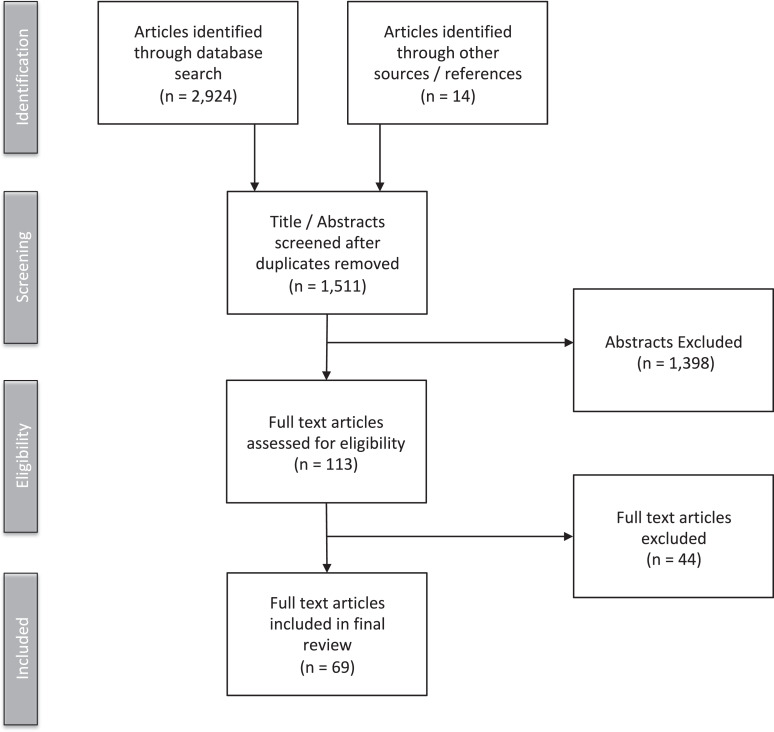
Procedure for study selection adapted from the PRISMA flow diagram.

The electronic search yielded 2,924 articles; an additional 14 articles were identified through references from selected articles. After duplicates were removed, 1,511 articles remained. Following screening of titles and abstracts, 1,398 articles were excluded. 113 full text articles were assessed for eligibility and 44 were excluded (n = 4 excluded for no access). 69 full text articles were included in the final review (Figure 1). Quantitative (n = 53), qualitative (n = 13) and mixed methods (n = 3) studies were included (Appendix E). The characteristics of the included tables are included in Table 1.

**Table 1.** Characteristics of identified studies.

Study Characteristics	Number of Included Studies (n = 69)
Parent Focus	
Mothers	28
Fathers	1
Mixed	35
Not Specified	5
Children Gender	
Males	4
Females	8
Mixed	49
Not Specified	8
Age of Child	
18 and younger	21
19 and older	1
Mixed ages	39
Not Specified	8
Country of Data Collection*	
United States	33
Canada	3
United Kingdom	10
Ireland	3
Europe	21
Australia	3
Asia	2
Other (Serbia, South Africa)	3

* Some studies took place in multiple countries.

### Measures of parental stress and adjustment

The majority of quantitative studies used measures of stress (e.g. Parenting Stress Index, Questionnaire on Resources and Stress; n = 34), mental health (e.g. Centre for Epidemiologic Studies–Depression Scale, Symptom Checklist-90; n = 28) and/or caregiver burden/strain (e.g. Caregiver Burden Index, Caregiver Strain Questionnaire; n = 5) to reflect adverse parental outcomes. Direct measures of positive affect or positive aspects of having a child with a genetic syndrome (e.g. Positive Gain Scale, Positive Affect Scale, Benefit Finding subscale; n = 10), quality of life (e.g. SF-36/12 Health Survey; n = 8), or wellbeing, life satisfaction and, psychological adaptation (e.g. Caregiver Well-Being Scale, Satisfaction with Life Scale, Psychological Adaptation Scale; n = 7) were used less frequently. As such, many studies imply that the absence of negative outcomes such as stress, mental health difficulties or burden is equivalent to wellbeing. Multiple measures were used to represent parental outcomes in several studies thus the total number of measures used is not equivalent to the number of studies explored in this review. Qualitative studies (n = 13) were also included to provide a deeper understanding of factors associated with parental stress and adjustment in the context of having a child with a rare genetic syndrome.

### Parental outcomes in rare genetic syndromes

Studies explored the prevalence of clinically significant parental outcomes relative to population norms, parents of typically developing children, Down syndrome and other disabilities. Within rare genetic syndromes, prevalence was also reported. Relative to population norms or typically developing populations, parental stress, psychological difficulties was significantly higher across several syndromes including Prader-Willi, Cornelia de Lange, Angelman, Cri du Chat, Williams and Fragile X syndromes (Griffith et al., 2011b; Mazaheri et al., 2013; Sarimski, 1997a, 1997b, 2010; Wheeler et al., 2018). Lower physical and emotional quality of life was also reported in Tuberous Sclerosis complex and Rett syndromes compared with normed data (Laurvick et al., 2006; Rentz et al., 2015). In these studies, stress related to the child domain or child characteristics was greater though the parent domain or parent domain subscales were also significant (Sarimski, 1997a, 1997b). A similar pattern was observed in comparative studies with typically developing populations. Higher parental stress in Williams and Fragile X syndromes relative to parents of typically developing children (Papaeliou et al., 2012; von Gontard et al., 2002; Zyga and Dimitropoulos, 2020), primarily driven by stress in the child domain.

Given its prevalence, Down syndrome was used as a comparative group in a number of studies and different profiles of stress were observed across syndromes. Greater parental stress was reported in Smith-Magenis, Williams, Prader-Willi and Fragile X syndromes relative to Down syndrome (Fidler et al., 2000; Hartley et al., 2012; Lanfranchi and Vianello, 2012; Lewis et al., 2006). Stress associated with child characteristics or difficulties was identified in Williams syndrome (Lanfranchi and Vianello, 2012; Papaeliou et al., 2012) but no difference in stress related to parent domain in Williams syndrome in comparison to Down syndrome (Ashworth et al., 2019; Papaeliou et al., 2012). However, parents of children with Cornelia de Lange syndrome indicated significantly higher parent distress rather than difficulties associated with the child relative to Down syndrome (Richman et al., 2009). Despite the differences in parental stress across multiple syndromes, no difference in mental health outcomes were observed in Fragile X and Cornelia de Lange syndromes relative to Down syndrome (Hartley et al., 2012; Lewis et al., 2006; Richman et al., 2009). Furthermore, no difference in life satisfaction between Fragile X and Down syndrome was observed (Lewis et al., 2006).

Exploration of parental stress and wellbeing in rare genetic syndromes was contextualised in relation to other disabilities including physical disability, intellectual disability and Autism Spectrum Disorder (ASD). Hodapp and colleagues reported high levels of parental stress in Smith-Magenis and Prader-Willi syndromes relative to intellectual disabilities, particularly in parents of children under 11 years (Hodapp et al., 1997, 1998). Higher parental stress in Fragile X syndrome and lower quality of life in Prader-Willi syndrome relative to complex chronic or physical conditions with the exception of physical incapacitation was also noted (Mazaheri et al., 2013; von Gontard et al., 2002). With regard to ASD, mothers reported higher stress in Angelman syndrome relative to ASD (Griffith et al., 2011b). In this study, fathers of children with Angelman or Cri du Chat syndromes were also more likely to reported clinical depression relative to fathers of children with ASD (Griffith et al., 2011b) however the opposite was true in fathers of Fragile X syndrome (Hartley et al., 2012). A number of studies demonstrated comparable or better outcomes in rare syndromes relative to ASD; for example, higher parental life satisfaction in Williams syndrome relative to ASD was highlighted (Ashworth et al., 2019). Furthermore, comparable parental outcomes were demonstrated between ASD, Williams syndrome and Fragile X syndromes (Ashworth et al., 2019; Chan et al., 2017; Hartley et al., 2012).

Syndrome-specific studies and comparative studies of rare genetic syndromes demonstrate variable prevalence rates of parent outcomes across syndromes. Rates of clinical stress ranged from over 50% (e.g. Cornelia de Lange syndrome, Angelman syndrome) to less than 30% (e.g. Fragile X syndrome, Prader-Willi syndrome) (Bailey et al., 2008; Baker et al., 2012; Wulffaert et al., 2009, 2010). Angelman syndrome was reported to have higher rates of parental stress relative to Prader-Willi, Cornelia de Lange and Cri du Chat syndromes (Griffith et al., 2011b; Wulffaert et al., 2010) while higher parental stress was noted in Prader-Willi relative to Williams syndrome (Lanfranchi and Vianello, 2012). In terms of mental health outcomes, over 85% of parents reported moderate to severe anxiety and/or depression in Smith-Magenis syndrome (Foster et al., 2010) while Rett and Barth syndromes reported clinical depression rates between 23% and 30% (Jacob et al., 2017; Sarajlija et al., 2013). If measured, the prevalence of anxiety was typically higher than depression within syndromes (Bailey et al., 2008; Foster et al., 2010; Jacob et al., 2017). Higher parental depression rates were reported in Williams syndrome relative to Fragile X syndrome (Sarimski, 1997a). Angelman and Cri du Chat syndromes also yielded higher rates of parental depression relative to Cornelia de Lange syndrome while higher anxiety rates were present in Angelman syndrome relative to Cornelia de Lange and Cri du Chat syndromes (Griffith et al., 2011b).

### Factors associated with parental stress and adjustment in rare genetic syndromes

In order to explore the factors associated with parental adversity and wellbeing in rare genetic syndromes, the findings across these syndromes are pooled and discussed in four sub-sections: (a) child-related factors, (b) parent-related factors, (c) family-related factors and, (d) contextual factors.

#### Child-related factors

##### Child demographics

A number of studies indicated that child age was not associated with parental stress (Byiers et al., 2014; Fidler et al., 2000; Sarimski, 2010; Wulffaert et al., 2010), life satisfaction (Shivers et al., 2016), strain (Jacob et al., 2017; Luescher et al., 1999) or quality of life (Laurvick et al., 2006) in a number of rare genetic syndromes. However, other studies demonstrated an association between increased child age and greater parent stress (Farmer et al., 2006; Hodapp et al., 1997; Sarimski, 1997b; Wulffaert et al., 2009). Killian and colleagues demonstrated that increased child age was associated with lower physical quality of life rather than emotional quality of life (Killian et al., 2016). Additionally, lower quality of life was experienced when parenting adolescents or premenarchal girls in Prader-Willi and Rett syndromes (Feighan et al., 2020; Ihara et al., 2014; Killian et al., 2016). A similar effect of age was observed in a longitudinal study of Rett syndrome. Lower emotional quality of life in parents when their children were between late childhood and late adolescence (Mori et al., 2019) but an increased emotional quality of life was observed in adulthood. This suggests different profiles of parental stress and wellbeing across the lifespan.

In terms of child gender, the majority of studies demonstrated no effect of gender on parental outcomes (Bailey et al., 2012; Baker et al., 2012; Briegel et al., 2008; Kopp et al., 2008; Shivers et al., 2016; Wulffaert et al., 2010; Wulffaert et al., 2009). Two studies demonstrated an effect of child gender. Higher paternal stress associated with girls in Joubert syndrome (Farmer et al., 2006) and parents of boys with Angelman syndrome had significantly higher rates of depression than parents of girls (Van Den Borne et al., 1999). In this study, mothers had significantly higher rates of depression than fathers thus these gender effects may also reflect relational parental differences.

##### Intellectual and adaptive functioning

Several studies indicated no impact of level of intellectual disability on parental outcomes in rare genetic syndromes (Briegel et al., 2007, 2008; Kopp et al., 2008; Shivers et al., 2016; von Gontard et al., 2002; Wulffaert et al., 2009). While Kopp and colleagues study of Tuberous sclerosis indicated no association between parental psychological difficulties and IQ, parental stress was associated with lower child IQ (Kopp et al., 2008). Furthermore, higher child domain stress was reported in parents of children with severe intellectual disability relative to moderate intellectual disability in Cornelia de Lange (Sarimski, 1997b). In Fragile X syndrome, child IQ was reported to have an indirect impact on maternal distress, mediated by child behaviour (Hall et al., 2007) thus the effects of intellectual disability on parental stress may be primarily due to the behaviours associated with the syndrome.

Studies exploring the impact of child adaptive functioning on parental outcome have produced mixed results. Adaptive functioning typically refers to abilities in four key areas: communication, daily living skills, socialisation, and motor skills. Parenting stress was associated with lower adaptive functioning in several studies where deficits in communication and socialisation were most commonly reported (Farmer et al., 2006; Hodapp et al., 1997, 1998; Kopp et al., 2008; Richman et al., 2009; Wulffaert et al., 2009; Zyga and Dimitropoulos, 2020). Several studies also reported no relationship between child adaptive functioning and parental outcomes (Bailey et al., 2008; Byiers et al., 2014; Griffith et al., 2011b; Luescher et al., 1999; Shivers et al., 2016; Smith et al., 2016). Total parental stress was not associated with degree of adaptive ability in Fragile X syndrome (Sarimski, 2010) though stress related to child difficulties was higher in parents of children with lower adaptive functioning. This suggests that lower adaptive functioning may exacerbate parental stress due to child behavioural difficulties.

##### Emotional and behavioural difficulties

Several instruments were used across studies to assess general behaviours (e.g. Child Behaviour Checklist, Developmental Behaviour Checklist, Aberrant Behaviour Checklist, Behaviour Assessment System for Children) relative to parental outcomes. Subscales on parental measures of stress (e.g. Difficult child on the PSI-4, Child domain on the PSI-4, Child characteristics on the QRS-F) also reflect child behaviours. Maladaptive behaviours were consistently associated with parental outcome measures. Both externalising behaviour, i.e. aggression, challenging behaviour (Adams et al., 2018; Bailey et al., 2012; Briegel et al., 2008; Fidler et al., 2000; Hallberg et al., 2010; Hodapp et al., 1998; Jacob et al., 2017; Kopp et al., 2008; Morse et al., 2014; Papaeliou et al., 2012; Richman et al., 2009; Smith et al., 2016; von Gontard et al., 2002), and internalising behaviour, i.e. anxious, withdrawn, mood disturbance (Fidler et al., 2000; Hodapp et al., 1998; McCarthy et al., 2006; Mori et al., 2019; Morse et al., 2014; Smith et al., 2016), were associated with parental distress, some with a cumulative effect (Cianfaglione et al., 2015; Mori et al., 2018; Sarajlija et al., 2013; Wheeler et al., 2018). Parental stress was also predicted by child characteristics and child temperament in Prader-Willi and Fragile X syndromes (Lanfranchi and Vianello, 2012; McCarthy et al., 2006; Sarimski, 2010). Clinical levels of anxiety and depression were observed in a pooled group of parents of children with Angelman, Cornelia de Lange and Cri du Chat syndromes irrespective of challenging behaviour (Adams et al., 2018). Higher challenging behaviour at baseline predicted improved maternal depression over time (Hauser et al., 2014) thus suggesting the relationship between parent distress and child behaviour may be mitigated by other factors.

Parental wellbeing was also associated with reduced syndrome-specific behaviours such as idiosyncratic face movements in Rett syndrome (Laurvick et al., 2006) and hyperphagia in Prader-Willi syndrome (Shivers et al., 2016). Reilly and colleagues reported distinct challenges for parents across four syndromes. Parents primarily endorsed social skills in Fragile X syndrome, obsessions in Prader-Willi syndrome, excessive sociability in Williams syndrome and learning difficulties in 22q11.2 deletion syndrome as the most significant aspects of parenting (Reilly et al., 2015) thus highlighting the need for syndrome-specific considerations.

##### Physical health and genotype

Physical health and syndrome-specific features (e.g. Rett Syndrome Behaviour Questionnaire) were explored and reported less frequently. Parental outcomes were associated with sleep disturbance (Hodapp et al., 1998; Mori et al., 2018), seizure activity (Byiers et al., 2014; Killian et al., 2016; Kopp et al., 2008), low child health vulnerability (Foster et al., 2010), feeding and gastrointestinal difficulties (Killian et al., 2016), and physical limitations or recent fractures (Laurvick et al., 2006; McCarthy et al., 2006).

Maternal uniparental disomy (when both copies of a chromosome are maternally inherited) has been found to be associated with poorer parental outcomes in Prader-Willi and Angelman syndromes (Ihara et al., 2014; Miodrag and Peters, 2015) relative to parents whose children have deletions. However, no association between genotype and parental outcomes have been observed in Rett syndrome (Laurvick et al., 2006) or Tuberous Sclerosis complex (Kopp et al., 2008).

#### Parent-related factors

##### Parent demographics

Variable patterns of association between parental demographics (age, gender, education) and parental outcomes were demonstrated across studies. Some studies indicated that parental age was not associated with parental outcomes (Fidler et al., 2000; Laurvick et al., 2006; Shivers et al., 2016). However, studies of parents of children with Rett syndrome demonstrated reduced physical quality of life with increased parental age (Killian et al., 2016; Mori et al., 2018, 2019). Increased emotional quality of life with increased parental age (Killian et al., 2016) as well as no association with emotional quality of life was also identified (Laurvick et al., 2006; Mori et al., 2018, 2019). Decline in physical quality of life was more significant when their children were in late childhood into adulthood (Mori et al., 2019).

Respondents were primarily mothers thus there was an imbalanced representation of parental gender. The majority of studies that included both mothers and fathers highlighted no significant difference between maternal and paternal outcomes (Byiers et al., 2014; Farmer et al., 2006; Kopp et al., 2008; Lanfranchi and Vianello, 2012; McCarthy et al., 2006) with the exception of one study which reported that depression was greater for mothers than fathers in both Prader-Willi and Angelman syndromes (van den Borne et al., 1999). McCarthy and colleagues highlighted that child internalising behaviours were associated with psychological distress in fathers of children with Fragile X syndrome but not mothers (McCarthy et al., 2006) thus child behaviour may differentially contribute to parental wellbeing.

The influence of education on parental outcomes was mixed. In some studies, a higher level of education was associated with greater parental wellbeing (Foster et al., 2010; Hodapp et al., 1998; Mori et al., 2018; Wheeler et al., 2018), however no association between education and parental outcomes was identified in other studies (Laurvick et al., 2006; Shivers et al., 2016).

##### Parental mental health, appraisal and coping

A number of studies demonstrated that parental outcomes measures were correlated. For example, psychological distress including anxiety and depression was associated with parental stress (Johnson et al., 2006; McCarthy et al., 2006), strain (Jacob et al., 2017), quality of life (Sarajlija et al., 2013) and wellbeing (Foster et al., 2010) across syndromes thus demonstrating concurrent interaction effects of these parental factors.

Parental perspectives with regard to their role as a parent and with regard to having a child with a rare genetic condition also influenced parental adjustment. Perceived parental self-efficacy, benefit finding, parenting knowledge and satisfaction in the caregiver role were associated with better outcomes (Foster et al., 2010; Lamb et al., 2016; Raspa et al., 2014). Benefit finding with regard to having a child with a rare genetic syndrome is captured more frequently in qualitative studies. In these studies, joy, pride and the positive impact of their child on their life perspective as well as their personal growth in terms of empathy, patience, humility and gratitude was illustrated (Goodwin et al., 2017; Scallan et al., 2011). On the other hand, parental perception of an external locus of control i.e. parents perception that children have control over their lives predicted parental stress, particularly parent and family problems (Lanfranchi and Vianello, 2012). Qualitative studies reflected this loss of control, a life centred round their child’s needs as well as a loss of self-identity in the caregiving role (Goodwin et al., 2017; Graffigna et al., 2013; Scallan et al., 2011).

Parent use of coping strategies was associated with stress (Jacob et al., 2017; von Gontard et al., 2002; Wheeler et al., 2018), strain (Jacob et al., 2017), mental health difficulties (Jacob et al., 2017; Luescher et al., 1999; Wheeler et al., 2018), psychological adaptation (Lamb et al., 2016) and maternal life satisfaction (Shivers et al., 2016). Jacob et al. (2017) highlighted differential associations between parental coping and parental outcomes. Jacob and colleagues demonstrated that behavioural disengagement and humour predicted anxiety; humour also predicted stress while self-blame predicted caregiver strain in Barth syndrome. Qualitative studies describe the need to take action, be proactive and plan for predictability to avoid triggers (Nag et al., 2019; Vitale, 2016). Passive appraisal, acquisition of social support, active coping, acceptance, flexibility and mindfulness were associated with lower stress, anxiety and depression (von Gontard et al., 2002; Wheeler et al., 2018) while maladaptive coping strategies such as avoidance, self-blame, wishful thinking, behavioural disengagement and increased substance use were associated with poorer parental outcomes (Jacob et al., 2017; Luescher et al., 1999; Shivers et al., 2016).

#### Family related factors

##### Family composition

Marital status was not associated with maternal life satisfaction or physical quality of life (Laurvick et al., 2006; Shivers et al., 2016), however higher physical quality of life and lower depression was associated with married status in Rett and Fragile X syndromes (Lebel et al., 2008; Mori et al., 2019; Wheeler et al., 2018). It must be noted that the unmarried parents were underrepresented at 19% or less in these studies. Parents described that caregiving demands and partners lack of understanding or support in managing these demands was often a key reason for relationship breakdown (Williamson, 2019). No association between number of siblings and parental wellbeing was observed in some studies (Kopp et al., 2008; Laurvick et al., 2006). However, other studies demonstrated that greater life satisfaction was positively associated with having more children in William Syndrome (Ashworth et al., 2019) while reduced parental wellbeing was associated with their child having two of more siblings in Rett Syndrome (Mori et al., 2018) or if they had an additional children with a disability (Hartley et al., 2012).

##### Family functioning

A number of measures of family functioning, support, coping and cohesion (e.g. Family Environment Scale, Family Assessment Device, Family Support Scale) assessed the influence of family factors. Parental wellbeing was promoted by greater family support (von Gontard et al., 2002) and marital adjustment (Baker et al., 2012; Laurvick et al., 2006; McCarthy et al., 2006; van Lieshout et al., 1998; Vitale, 2016) across syndromes. Family functioning, family dynamics and the family environment were also associated with emotional quality of life and psychological adaptation (Killian et al., 2016; Lamb et al., 2016; Mori et al., 2019), caregiver strain (Luescher et al., 1999), and parental stress (Hall et al., 2007). Family functioning also had a mediating effect on the relationship between parental stress and child behaviour (Morse et al., 2014) as well as between parental adaptation and coping (Lamb et al., 2016). Family adaptability and cohesion also predicted stress and maternal internalising symptoms (Baker et al., 2012; Johnson et al., 2006; Lanfranchi and Vianello, 2012). Vitale also described how striving for family agreement and cohesion promoted family functioning and improved general parental wellbeing (Vitale, 2016).

#### Contextual factors

Positive parental outcomes were related to non-familial support from friends or other parents (Hodapp et al., 1998; Palacios-Cena et al., 2018; Raspa et al., 2014) and health care professionals (Foster et al., 2010; Hallberg et al., 2010). Lack of time resources (Killian et al., 2016; Laurvick et al., 2006; Mori et al., 2018; Rentz et al., 2015; Williamson, 2019) and higher caregiving hours (Bailey et al., 2012) was associated with poorer parental quality of life and wellbeing. Qualitative studies offered greater contextual information regarding the interaction between systemic issues and parental wellbeing. Several studies highlighted frustration and despair associated with the lack of professional knowledge, difficulty accessing services and lack of awareness of rare genetic syndromes within the system (Cagalj et al., 2018; Goodwin et al., 2017; Griffith et al., 2011a; Hallberg et al., 2010; Nag et al., 2019; Scallan et al., 2011).

Contextual factors such as employment, financial and residential status and their association with parental wellbeing were also explored. Due to caregiving demands, parents reported challenges with employment (Feighan et al., 2020; Palacios-Cena et al., 2018). Parents in employment (Laurvick et al., 2006; Mori et al., 2018; Sarajlija et al., 2013) or low financial strain (Bailey et al., 2012; Johnson et al., 2006; Laurvick et al., 2006; Mori et al., 2018; Raspa et al., 2014) typically demonstrated lower burden and stress and greater wellbeing. The relationship between parental outcomes and residential status was complex. No relationship was observed with child residential status in two studies (Baker et al., 2012; Byiers et al., 2014). While having their child living at home was associated with lower physical quality of life (Mori et al., 2019) and had a greater emotional impact (Feighan et al., 2020), respite was also associated with a lower emotional quality of life (Mori et al., 2019). Furthermore, having their child living at home was a predictor of positive gains in parents of children with a genetic syndrome (Cianfaglione et al., 2015).

## Discussion

The aim of this review was to explore parental stress and adjustment in the context of rare genetic syndromes and examine factors associated with parental outcomes in order to highlight gaps in knowledge, to promote parent adjustment and improve the provision of family-centred care. The first key finding demonstrated that a significant proportion of parents with children diagnosed with a rare genetic syndrome experienced clinically significant levels of stress, strain and mental health difficulties relative to population norms, parents of typically developing children, parents of children with Down syndrome and parents of children with a physical disability or an intellectual disability of unknown aetiology. However, relative to ASD, the findings were more variable dependent on the syndrome. The prevalence of ASD has been estimated at approximately 1 in 68 (Christensen et al., 2016) thus occurring more frequently than rare genetic syndromes. These results suggest that a defined genetic aetiology or the uncommon nature of these syndromes alone do not account for differential parental outcomes in rare genetic syndromes.

Furthermore, significant variation on parental outcome measures was observed across rare genetic syndromes. Syndromes such as Angelman, Cornelia de Lange and Smith-Magenis syndrome reported higher prevalence of parental adversity relative to norms and relative to other syndromes. This suggests that syndrome-specific phenotypes likely contribute to parent stress and adjustment. The importance of understanding the behavioural phenotypes of genetic syndromes associated with intellectual disability has previously been argued (Tunnicliffe and Oliver, 2011; Waite et al., 2014). Waite highlighted that some practitioners have rejected diagnostic genetic syndromes labels as they emphasise that the medical model of understanding human difficulties is irrelevant to individuals with an intellectual disability. On the other hand, Waite suggests that knowledge of behavioural phenotypes may be useful to anticipate difficulties, improve behavioural formulation and promote the wellbeing of the individual (Waite et al., 2014). This review suggests that it may be useful to incorporate rare syndromic diagnoses and their associated phenotype into the formulation of family difficulties due to an elevated risk of parental distress in these conditions.

A number of factors were shown to influence parental stress in the context of their child’s genetic condition. The findings demonstrated that both child maladaptive behaviour and child emotional difficulties consistently predicted poorer parental outcomes across rare genetic syndromes. This association is consistent with existing disability literature (McStay et al., 2014; Salomone et al., 2018; Yorke et al., 2018). Significantly, the majority of studies demonstrated that level of intellectual functioning was not associated with parental outcomes but it indirectly mediated the relationship between parent stress and child behaviour. Furthermore, it appears that adaptive functioning, particularly communication and socialisation skills, are more likely to have an impact on parental outcomes, both directly and indirectly. These findings suggest that it is the level of associated adaptive and behavioural challenges rather than the level of intellectual disability that predominantly impacts parental wellbeing. This may have significant implications within decision-making process for care provision, particularly in the case of individuals whose genetic syndrome is characterised by a mild intellectual disability, i.e. a mild intellectual disability is not necessarily equivocal to mild needs, other behaviours must also be considered.

Interestingly, late childhood into early adulthood emerged as a potential risk period for parental adversity. Quality of life studies indicate that physical rather than emotional parental wellbeing declines with both child and parental age at this time. This suggests that the physical demands of caregiving are a significant factor. This is supported by the association between lack of time resources, higher caregiving hours and poorer parental outcomes identified in this review. It is likely that a number of child and contextual factors contribute to a greater burden of stress during this period. It has been highlighted that maladaptive behaviours increased during adolescence in both disability and neurotypical populations (Brereton et al., 2006; VanderValk et al., 2005). Furthermore, the transition from childhood to adulthood services has also been highlighted as a significant period of distress in disability literature (Henninger and Taylor, 2014; Neece et al., 2009) thus anticipation of transition, coupled with uncertain prognostic outcomes for parents of children with a rare condition may exacerbate parental distress during this period. Higher emotional quality of life was also reported after their child reached adulthood which may reflect increased parental adaptation and reduced concerns about the future welfare of their child.

To date, much of the research has predominantly focused on how child-related factors impact parental stress and adjustment. Parental, family and contextual related factors have been explored in the research to a lesser extent. Parental stress or caregiver burden measures were correlated with mental health measures, thus illustrating a reciprocal relationship between these factors in genetic syndromes. This review also demonstrated differential relationships between parental coping strategies and measures of parental stress or mental health difficulties suggesting that certain coping styles may have better outcomes depending on type of burden experienced by parents. Both adaptive and maladaptive coping strategies are evidenced highlighting that parents vary in their ability to cope and manage their child’s care needs in these syndromes. Self-efficacy and cognitive appraisal have previously been shown to mediate the effect of child behavioural difficulties on parent stress in both ASD and developmental difficulties (Hastings and Brown, 2002; Plant and Sanders, 2007). This review indicates that a similar benefit may be observed in rare genetic syndromes. Several family factors including functioning, cohesion, support and the environment were associated with greater parental adjustment. Family functioning was also shown to mediate the relationship between parental stress and child behaviour. The findings suggest that family factors have both a direct and an indirect impact on parental wellbeing in the context of rare genetic syndromes. Furthermore, family functioning has also shown to have a mediating effect between parental adjustment and parental self-efficacy, emotion-focused and problem focused coping. This demonstrates a presence of complex interactions between parental appraisal, parental coping, family functioning and parental wellbeing. It has been illustrated that addressing parental psychological functioning is beneficial for their child (Law et al., 2019) thus demonstrating the need for consider family-centred care. Consistent with previous disability literature, greater income, time available, and access to social support were positively associated with parental wellbeing in these syndromes (Smith et al., 2001).

### Gaps in the literature

This review highlighted a number of gaps in the literature and considerations for future research. Genetic syndromes are often reported to bring specific physical needs or complex medical conditions however, the impact of such conditions have not been routinely explored in rare genetic syndrome literature. Rett syndrome is a possible exception due the development of a Rett specific behaviour-related questionnaire. While some studies demonstrated an association between health conditions and poor parental outcomes, a number of studies excluded any measure of health or physical limitations. In addition, despite the likely contribution of syndrome-specific phenotype and behaviour to parental distress, few studies specifically explored behaviours directly associated with the studied syndrome, e.g. hyperphagia in Prader-Willi syndrome. This review highlights the need to develop and incorporate syndrome-specific measures into future research in order to understand how syndrome-associated behaviour and health conditions contribute to parental distress. While it is apparent that some aspects of the impact of having a child with a rare genetic syndrome are similar, there are also likely to be syndrome-specific aspects that are more pertinent for particular syndromes.

Furthermore, findings in this review preliminarily suggest that the factors associated with parental stress and adjustment may be differentially associated between mothers and fathers. However, even in mixed parent gender studies, the respondents were predominantly mothers. Thus, future studies should purposefully seek out fathers as respondents in order to explore any relational differences in parental wellbeing. A greater depth of research is required to evaluate parental appraisal, coping and adjustment in rare genetic conditions. In particular, benefit finding in the parental experience as well as the role of uncertainty and sense of agency or self-efficacy in the context of a genetic aetiology should be explored further. Few studies included measures of positive aspects or wellbeing and all, with the exception of one study, explored them in the context of mitigating stress rather than as outcome measures. Thus, in the current literature, parental adjustment is implied by the absence of negative outcomes rather than by the presence of factors associated with wellbeing such as autonomy, self-acceptance, purpose in life, environmental mastery, positive relationships, personal growth that interact and contribute to psychological wellbeing (Ryff, 2014).

### Clinical implications

The current review has significant clinical relevance in the context of disability services and family wellbeing. Firstly, it demonstrates the need to look beyond the level of intellectual functioning when determining allocation of family resources in these conditions and consider other child factors such as internalising and externalising difficulties, adaptive behaviour, physical health as well as syndrome-specific features. McConkey (2005) demonstrated that provision of care was typically decided based on the characteristics of the individual with a disability and highlighted the need to also consider family characteristics in the provision of care. This review supports that stance and illustrates the need to incorporate parental and family functioning, their financial situation and support structure in the decision-making process for individuals with rare genetic syndromes. At a systemic level, increased supports and resources for parents should be made available during the late childhood-early adulthood period in order to support parents’ psychosocial adjustment to their child’s needs.

### Strengths and limitations

A strength of this study is the systematic and broad search that likely captured all relevant articles published in academic journals. The search did not address grey material, a factor that may have resulted in missing relevant information. In addition, the absence of a quality assessment prior to study inclusion must also be acknowledged. As such, some of the conclusions drawn may be considered tentative. However, quality evaluation of studies is not typically conducted in scoping reviews (Peterson et al., 2017) due to the large variety of study designs, research approaches and the relatively new and emerging focus of the study. This project, therefore, focused on the information provided within the studies rather than obtaining information after a quality-based selection. Finally, for the purpose of this review, factors associated with parental outcomes were not considered within rare genetic syndromes. This decision was justified given the current paucity of family research within a number of these syndromes.

## Conclusion

This review aimed to explore parental stress and adjustment in rare genetic syndromes to highlight the current gaps in knowledge, to promote parent adjustment and improve the provision of family-centred care. Findings highlighted that parents of children with rare genetic syndromes experience greater distress relative to parents of children with Down syndrome or with intellectual disabilities of unknown aetiology. Differences in parental wellbeing were syndrome-specific relative to ASD thus demonstrating the need to consider syndrome-specific phenotypes. The relationship between parental outcomes and child emotional and behavioural difficulties was the most consistent finding across studies. Research reflecting other factors such as physical health, syndrome-specific behaviours, benefit finding and, parental appraisal and coping in the context of a rare genetic aetiology is required in order to support parental adjustment in these conditions.

## Supplemental material

Supplemental Material, sj-docx-1-jld-10.1177_1744629521995378 - Parental stress and adjustment in the context of rare genetic syndromes: A scoping review Click here for additional data file. Supplemental Material, sj-docx-1-jld-10.1177_1744629521995378 for Parental stress and adjustment in the context of rare genetic syndromes: A scoping review by Jacqueline Fitzgerald and Louise Gallagher in Journal of Intellectual Disabilities
