# Supplementary material for: Parental stress and adjustment in the context of rare genetic syndromes: A scoping review
Source: J Intellect Disabil. 2021 Apr 19;26(2):522–44. doi: 10.1177/1744629521995378 (PMC9168905; doi:10.1177/1744629521995378)
Supplement: Supplemental Material, sj-docx-1-jld-10.1177_1744629521995378 - Parental stress and adjustment in the context of rare genetic syndromes: A scoping review [file sj-docx-1-jld-10.1177_1744629521995378.docx]

| Author | Syndrome | Title |
| --- | --- | --- |
| Adams et al., 2018 | Angelman  Cornelia de Lange  Cri Du Chat | Mental health and well-being in mothers of children with rare genetic syndromes showing chronic challenging behavior: A cross-sectional and longitudinal study |
| Ashworth et al., 2019 | Williams | Comparing parental stress of children with neurodevelopmental disorders: The case of Williams syndrome, Down syndrome and autism spectrum disorders |
| Bailey et al., 2008 | Fragile X | Child and Genetic Variables Associated with Maternal Adaptation to Fragile X Syndrome: A Multidimensional Analysis |
| Bailey et al., 2012 | Fragile X | Health and Economic Consequences of Fragile X Syndrome for Caregivers |
| Baker et al., 2012 | Fragile X | Behaviour problems, maternal internalising symptoms and family relations in families of adolescents and adults with fragile X syndrome |
| Briegel et al., 2007 | 22q | 22q11.2 deletion syndrome: behaviour problems of infants and parental stress |
| Briegel et al., 2008 | 22q | 22q11.2 deletion syndrome: behaviour problems of children and adolescents and parental stress |
| Byiers et al., 2013 | Rett | Seizures and pain uncertainty associated with parenting stress and Rett syndrome |
| Cagalj et al., 2018 | Prader-Willi | Being a Mother of a Child with Prader-Willi Syndrome: Experiences of Accessing and Using Formal Support in Croatia. |
| Chan et al., 2017 | Fragile X | Executive Functioning Mediates the Effect of Behavioral Problems on Depression in Mothers of Children With Developmental Disabilities |
| Cianfaglione et al., 2015 | Rett | Psychological well-being of mothers and siblings in families of girls and women with Rett syndrome |
| Cianfaglione et al., 2017 | Rett | Change over a 16-month period in the psychological well-being of mothers of girls and women with Rett syndrome |
| Farmer et al., 2006 | Joubert | Parenting Stress and Its Relationship to the Behaviour of Children with Joubert Syndrome |
| Feighan et al., 2020 | Prader-Willi | A Profile of Mental Health and Behaviour in Prader-Willi Syndrome |
| Fidler et al., 2000 | Smith-Magenis  Williams | Stress in families of young children with Down syndrome, Williams syndrome, and Smith-Magenis syndrome. |
| Fong et al., 2019 | Tuberous Sclerosis Complex | Quality of life of children with tuberous sclerosis complex. |
| Foster et al., 2010 | Smith-Magenis | Caring for the caregivers: An investigation of factors related to well-being among parents caring for a child with Smith-Magenis syndrome. |
| Goodwin et al., 2017 | 22q | "You don't know until you get there": The positive and negative "lived" experience of parenting an adult child with 22q11.2 deletion syndrome. |
| Graffigna et al., 2013 | Tuberous Sclerosis Complex | Assisting a child with tuberous sclerosis complex (TSC): a qualitative deep analysis of parents’ experience and caring needs |
| Griffith et al., 2011 | Angelman  Cornelia de Lange  Cri Du Chat | Psychological well‐being in parents of children with Angelman, Cornelia de Lange and Cri du Chat syndromes. |
| Griffith et al., 2011 | Angelman  Cornelia de Lange  Cri Du Chat | 'You have to sit and explain it all, and explain yourself.' Mothers’ experiences of support services for their offspring with a rare genetic intellectual disability syndrome. |
| Hall et al., 2007 | Fragile X | Modeling family dynamics in children with fragile X syndrome. |
| Hallberg et al., 2010 | 22q | 22q11 deletion syndrome—The meaning of a diagnosis. A qualitative study on parental perspectives. |
| Hartley et al., 2012 | Fragile X | Psychological well‐being in fathers of adolescents and young adults with Down Syndrome, Fragile X syndrome, and autism. |
| Hauser et al., 2014 | Fragile X | Maternal well-being and child behavior in families with fragile x syndrome. |
| Hodapp et al., 1997a | Prader-Willi | Families of children with Prader-Willi syndrome: Stress-support and relations to child characteristics. |
| Hodapp et al., 1998 | Smith-Magenis | Stress and coping in families of children with Smith–Magenis syndrome. |
| Ihara et al., 2014 | Prader-Willi | QOL in caregivers of Japanese patients with Prader-Willi syndrome with reference to age and genotype |
| Jacob et al., 2017 | Barth | Psychosocial functioning in Barth syndrome: Assessment of individual and parental adjustment |
| Johnston et al., 2003 | Fragile X | Factors Associated with Parenting Stress in Mothers of Children with Fragile X Syndrome. |
| Killian et al., 2016 | Rett | Caretaker Quality of Life in Rett Syndrome: Disorder Features and Psychological Predictors. |
| Kopp et al., 2008 | Tuberous Sclerosis Complex | Behavior problems in children with tuberous sclerosis complex and parental stress |
| Lamb et al., 2016 | Rett | Family functioning mediates adaptation in caregivers of individuals with Rett syndrome. |
| Lanfranchi & Vianello, 2012 | Prader-Willi  Williams  Fragile X | Stress, locus of control, and family cohesion and adaptability in parents of children with Down, Williams, Fragile X, and Prader-Willi syndromes. |
| Laurvick et al., 2006 | Rett | Physical and mental health of mothers caring for a child with Rett syndrome. |
| Lewis et al., 2006 | Fragile X | Psychological well-being of mothers of youth with fragile X syndrome: Syndrome specificity and within-syndrome variability |
| Luescher et al., 1999 | Joubert | Parental Burden, Coping, and Family Functioning in Primary Caregivers of Children with Joubert Syndrome |
| Mazaheri et al. 2013 | Prader-Willi | The impact of Prader—Willi syndrome on the family's quality of life and caregiving, and the unaffected siblings’ psychosocial adjustment. |
| McCarthy et al., 2006 | Fragile X | Predictors of stress in mothers and fathers of children with fragile X syndrome. |
| Miodrag and Peters, 2015 | Angelman | Parent stress across molecular subtypes of children with Angelman syndrome. |
| Mori et al., 2018 | Rett  CDLK5 | Comparing parental well-being and its determinants across three different genetic disorders causing intellectual disability. |
| Mori et al., 2019 | Rett | Longitudinal effects of caregiving on parental well-being: the example of Rett syndrome, a severe neurological disorder. |
| Morse et al., 2014 | Smith-Magenis | Effects of behavior problems, family functioning, and family coping on parent stress in families with a child with Smith-Magenis Syndrome. |
| Nag et al., 2019 | Smith-Magenis | Parental experiences with behavioural problems in Smith–Magenis syndrome: The need for syndrome-specific competence. |
| Palacios-Cena et al., 2018 | Rett | "Living an Obstacle Course": A Qualitative Study Examining the Experiences of Caregivers of Children with Rett Syndrome. |
| Papaeliou et al., 2012 | Williams | Behavioural profile and maternal stress in Greek young children with Williams syndrome. |
| Raspa et al., 2014 | Fragile X | Modeling family adaptation to Fragile X syndrome. |
| Reilly et al., 2015 | 22q  Prader-Willi  Williams  Fragile X | The Impact on the Family of Four Neurogenetic Syndromes: A Comparative Study of Parental Views. |
| Rentz et al., 2015 | Tuberous Sclerosis Complex | Caring for Children With Tuberous Sclerosis Complex: What Is the Physical and Mental Health Impact on Caregivers? |
| Retzlaff et al., 2007 | Rett | Families of Children With Rett Syndrome: Stories of Coherence and Resilience |
| Richman et al., 2009 | Cornelia de Lange | Parenting stress in families of children with Cornelia De Lange syndrome and down syndrome. |
| Sarajlija et al., 2013 | Rett | Health-related quality of life and depression in Rett syndrome caregivers |
| Sarimski et al., 1997a | Prader-Willi  Williams  Fragile X | Behavioural phenotypes and family stress in three mental retardation syndromes. |
| Sarimski, 1997b | Cornelia de Lange | Communication, social-emotional development and parenting stress in Cornelia-de-Lange syndrome. |
| Sarimski, 2010 | Fragile X | Adaptive skills, behavior problems, and parenting stress in mothers of boys with Fragile X syndrome. |
| Scallan et al., 2011 | Williams | Williams syndrome: Daily challenges and positive impact on the family. |
| Shivers et al., 2016 | Prader-Willi | Life satisfaction among mothers of individuals with Prader-Willi syndrome. |
| Smith et al., 2016 | Fragile X | Change in the Behavioral Phenotype of Adolescents and Adults with FXS: Role of the Family Environment |
| Thomson et al., 2017 | Prader-Willi  Angelman | “Over time it just becomes easier...”: parents of people with Angelman syndrome and Prader–Willi syndrome speak about their carer role |
| VanDenBorne et al., 1999 | Prader-Willi  Angelman | Psychosocial problems, coping strategies, and the need for information of parents of children with Prader-Willi syndrome and Angelman syndrome. |
| VanLieshout et al., 1998 | Prader-Willi  Williams  Fragile X | Family contexts, parental behaviour, and personality profiles of children and adolescents with Prader-Willi, fragile-X, or Williams syndrome. |
| Vitale et al., 2015 | Prader-Willi | Parent Recommendations for Family Functioning With Prader-Willi Syndrome: A Rare Genetic Cause of Childhood Obesity. |
| VonGontard et al. 2002 | Fragile X | Psychopathology and familial stress comparison of boys with Fragile X syndrome and Spinal Muscular Atrophy |
| Weber et al., 2016 | Fragile X | Understanding fragile X syndrome from a mother’s perspective: Challenges and resilience |
| Wheeler et al., 2018 | Fragile X | Mindfulness and Acceptance as Potential Protective factors for mothers of children of Fragile X |
| Williamson, 2018 | Rett | ‘*I am everything but myself*’: Exploring visual voice accounts of single mothers caring for a daughter with Rett syndrome. |
| Wulffaert et al., 2009 | Cornelia de Lange | Simultaneous analysis of the behavioural phenotype, physical factors, and parenting stress in people with Cornelia de Lange syndrome. |
| Wulffaert et al., 2010 | Prader-Willi  Angelman | Maternal parenting stress in families with a child with Angelman syndrome or Prader-Willi syndrome. |
| Zyga & Dimitropoulos, 2020 | Williams | Preliminary Characterization of Parent-Child Interaction in Preschoolers With Prader-Willi Syndrome: The Relationship Between Engagement and Parental Stress |
